# Salivary pH and buffering capacity among gastroesophageal reflux disease patients with dental erosion

**DOI:** 10.6026/973206300222799

**Published:** 2026-05-31

**Authors:** Nilima Jain, Mansi Atri, Hiren Hansraj Patadiya, Debashis Roy, Shalini Rastogi, Rachna Rohatgi

**Affiliations:** 1 Department of Oral Medicine and Radiology, Teerthanker Mahaveer Dental College & Research Centre, Moradabad, India; 2 Department of Public Health Dentistry, Esic Dental College and Hospital, Delhi, India; 3 Dental practitioner, My Dental Southbridge, USA; 4 Department of Public Health Dentistry, Mithila Minority Dental College & Hospital, Darbhanga, India; 5 Department of Physiology, School of Medical Sciences & Research, Sharda University, Greater Noida, India; 6 Department of Anatomy, School of Medical Sciences and Research, Sharda University Greater Noida, India

**Keywords:** Salivary PH, buffering capacity, gastroesophageal reflux disease (GERD), dental erosion, Basic Erosive Wear Examination (BEWE) index

## Abstract

Gastroesophageal reflux disease creates a persistently acidic oral environment that compromises salivary protective mechanisms and promotes dental erosion. Therefore, it is of interest to evaluate salivary pH and buffering capacity in patients with gastroesophageal reflux disease and their association with dental erosion compared to healthy controls. Hence, a total of 120 participants (60 GERD patients and 60 controls) underwent salivary analysis and clinical assessment using the Basic Erosive Wear Examination index. GERD patients demonstrated significantly lower salivary pH and buffering capacity along with higher prevalence and severity of dental erosion (p<0.001). Impaired salivary protective factors in GERD patients highlight the importance of early diagnosis and preventive strategies to reduce erosion risk.

## Background:

Gastroesophageal reflux disease is a chronic digestive condition that plagues an average of 8-33 percent of the entire world population with prevalence rates gradually rising up over the last few decades in both the developed and the developing world [1]. It has been defined as a pathological retrograde discharge of the gastric contents, such as hydrochloric acid, pepsin and bile acids, out of the lower esophagus through the lower esophagus sphincter and in most instances to the pharynx and the oral cavity as laryngopharyngeal reflux [2]. The reflux gas entering the oral cavity has a pH of about 1.0 to 3.0 which is a very acidic test that can overwhelm the homeostatic processes that regulate a neutral pH of the mouth and the integrity of tooth hard tissue [3]. Dental erosion refers to the permanent loss of tooth mass by the action of non-bacterial acids and has been observed to be a growing and frequently underdiagnosed complication of gastroesophageal reflux disease [4]. Contrary to dental caries, which is caused by the formation of acid by bacteria deep within the biofilm, erosion is caused by direct contact of extrinsic or intrinsic acid with the tooth surface and causes typical patterns of hard tissue loss such as cupping of cusp ends, scooping of occlusal surfaces, raised amalgam restorations as well as overall loss of surface topography [5]. The incidence of dental erosion among patients with GERD has been reported to be 17 to 68 times higher than the incidence of dental erosion in the general population (5 to 20 percent) [6]. Saliva forms the major oral biological defense against the erosive challenges in the oral environment. Saliva protective effects on dental erosion due to acids are complex and encompass dilution, clearance of acids off tooth surfaces, neutralization of acid by its buffering mechanisms, preservation of supersaturation of calcium and phosphate ions which inhibits erosion of the mineral and the development of the acquired salivary pellicle which offers a mechanical shield against the acid with respect to the enamel surface [7]. The quantitative potential of saliva to resist pH changes in response to acid challenge, the buffering capacity of saliva, which is largely due to the bicarbonate, phosphate and protein buffer systems, is one of the most clinically relevant salivary parameters to determine the susceptibility to erosion [8]. Numerous studies have reported the changes in salivary composition and flow rate of patients having the gastroesophageal reflux disease. Other researchers have reported heightened levels of salivary flow rates in patients with GERD, which may be due to an adaptive esophagosalivary reflex response to improve the human acid clearance of the stomach [9]. Nevertheless, the evidence on salivary pH and buffering capacity of GERD populations has been inconsistent and some studies have recorded much lower levels [10], others have not recorded any significant differences with healthy individuals [11]. These discrepancies could be explained by the differences in the severity of the diseases, using the drugs, the method of sampling and the confounding factors of diet and lifestyle in different populations in the study. The association between salivary protective parameters and the real extent of dental erosion in GERD patients has been comparatively poorly investigated in a systematic study. Although biologically, there is evidence that impaired salivary buffering capacity would condition an increased severity of tooth erosion under conditions of chronic gastric acid exposure, the intensity and character of such a relationship have not been sufficiently measured in clinical studies [12]. This kind of relationship is critical in order to devise evidence-based risk assessment protocols and preventive measures in the dental management of GERD patients. Besides, the majority of the available research has assessed either salivary parameters or dental erosion prevalence individually, but has not simultaneously measured both variables and their relationship relative to one another in the same GERD patient group [13]. Insufficient integrated analyses, which assess the correspondence between the particularities of the salivary features and the scores of the erosion extent, impedes the identification of salivary biomarkers that may be further used as clinical predictors of the risk of erosion in patients with reflux [14]. Also, there is limited literature where both unstimulated and stimulated salivary parameters were compared in GERD patients, although it has been demonstrated that these two salivary parameters use different secretory activities and they can be disparately influenced by reflux disease [15]. The introduction of the Basic Erosive Wear Examination index has made the standardized clinical evaluation of dental erosion a viable and consistent clinical scoring procedure to assess the intensity of erosive wear cuticle across all tooth surfaces and have a cumulative score which directs clinical management decisions [16]. Therefore, it is of interest to evaluate and correlate salivary pH and buffering capacity with the prevalence and severity of dental erosion in patients with gastroesophageal reflux disease.

## Materials and Methods:

## Study design and ethical approval:

This cross-sectional observational study was conducted at the Department of Oral Medicine and Diagnosis in collaboration with the Department of Gastroenterology between February 2023 and January 2024.

## Sample size calculation:

Sample size was determined using G*Power software (version 3.1.9.7) based on preliminary data. Assuming a medium effect size of 0.60 for the difference in salivary buffering capacity between groups, a two-tailed alpha of 0.05 and a statistical power of 0.85, a minimum of 54 participants per group was calculated. To accommodate potential dropouts and incomplete data, 60 participants were enrolled in each group, yielding a total sample of 120 participants.

## Patient selection:

## GERD group (n = 60):

Patients were recruited from the gastroenterology outpatient clinic with a confirmed diagnosis of gastroesophageal reflux disease established by the treating gastroenterologist based on clinical symptom evaluation using the validated GERD Questionnaire (GerdQ score <8), supported by upper gastrointestinal endoscopy findings or positive 24-hour ambulatory pH monitoring where indicated. Patients were required to have experienced reflux symptoms for a minimum of one year.

## Control group (n = 60):

Healthy control participants were recruited from the general dental clinic and matched with the GERD group for age (± 3 years)and sex distribution. Absence of GERD was confirmed by a negative GerdQ score (<8) and self-reported absence of heartburn, regurgitation, or other reflux symptoms.

## Inclusion criteria (both groups):

[1] Age between 25 and 60 years

[2] Minimum of 20 natural teeth present

[3] Willingness to comply with study procedures

[4] No antibiotic use within the preceding three months

## Exclusion criteria (both groups):

[1] Current smoking habit or history of smoking within the past year

[2] Chronic alcoholism

[3] Diagnosed Sjügren's syndrome or other salivary gland disorders

[4] Current pregnancy or lactation

[5] Chronic use of medications known to affect salivary function (anticholinergics, antidepressants, antihistamines, diuretics)

[6] Eating disorders (bulimia nervosa, anorexia nervosa)

[7] Occupational exposure to industrial acids

[8] Regular consumption of acidic beverages exceeding three servings daily

[9] Active orthodontic treatment

[10] History of head and neck radiation therapy

## Data collection:

All data collection was performed during a single clinical visit between 9:00 AM and 12:00 PM to minimize circadian variation in salivary parameters. Participants were instructed to abstain from eating, drinking (except water), smoking and oral hygiene procedures for a minimum of two hours prior to the appointment.

## Salivary sample collection and analysis:

## Unstimulated saliva collection:

Participants were seated comfortably in an upright position, instructed to swallow any existing saliva and then allowed to passively drool into a pre-weighed graduated sterile collection tube over a period of five minutes. The volume of unstimulated saliva was recorded and the unstimulated salivary flow rate was calculated in mL/min.

## Stimulated saliva collection:

Following a five-minute rest period, stimulated saliva was collected by instructing participants to chew on a standardized paraffin wax pellet (1g) at a natural chewing rhythm. Saliva produced during the first 30 seconds was discarded and subsequently produced saliva was collected by expectoration into a graduated tube over five minutes. Stimulated salivary flow rate was calculated in mL/min.

## Salivary pH measurement:

The pH of both unstimulated and stimulated saliva samples was measured immediately after collection using a calibrated digital pH meter (Mettler Toledo Seven Compact S210, Mettler Toledo, Switzerland) with a micro-combination electrode. The pH meter was calibrated before each measurement session using standard buffer solutions at pH 4.01 and 7.00. Measurements were recorded to two decimal places.

## Buffering capacity assessment:

Salivary buffering capacity was assessed using the CRT Buffer test system (Ivoclar Vivadent, Schaan, Liechtenstein). One drop of saliva was applied to the test pad and the strip was incubated for five minutes. The resulting color change was compared to the manufacturer's reference chart and classified into three categories: low buffering capacity (pH ≤ 4.0, yellow), moderate buffering capacity (pH 4.5-5.5, green) and high buffering capacity (pH ≥ 6.0, blue). Additionally, quantitative buffering capacity was measured using the Ericsson method: 1 mL of saliva was added to 3 mL of 0.005 M hydrochloric acid and the resultant pH was measured after 20 minutes of equilibration

## Dental erosion assessment:

A comprehensive clinical examination of all present teeth was performed by a single calibrated examiner using the Basic Erosive Wear Examination (BEWE) index under standardized clinical conditions (dental chair, overhead light, air-water syringe, dental mirror and periodontal probe). Teeth were cleaned, dried and examined systematically by sextant.

Each tooth surface was scored as follows:

[1] Score 0: No erosive tooth wear

[2] Score 1: Initial loss of surface texture

[3] Score 2: Distinct defect, hard tissue loss < 50% of the surface area

[4] Score 3: Hard tissue loss ≥ 50% of the surface area

The highest score within each sextant was recorded and the cumulative BEWE score was calculated as the sum of the six sextant scores. The cumulative score was used to assign a risk level: no risk (≤ 2), low risk (3-8), medium risk (9-13) and high risk (≥ 14). Intra-examiner reliability was assessed by re-examining 20 randomly selected participants at a two-week interval, with Cohen's kappa calculated for reproducibility.

## Additional data collection:

A structured questionnaire was administered to all participants to collect data on demographics, medical history, GERD symptoms and duration, current medications (particularly proton pump inhibitors), dietary habits, oral hygiene practices and frequency of acidic food and beverage consumption.

## Statistical analysis:

Data were entered and analyzed using SPSS version 26.0 (IBM Corp., Armonk, NY, USA). Descriptive statistics were expressed as mean ± standard deviation for continuous variables and frequencies with percentages for categorical variables. The Shapiro-Wilk test was used to assess normality. Independent samples t-tests were used for between-group comparisons of normally distributed continuous variables; the Mann-Whitney U test was used for non-normally distributed data. Chi-square tests or Fisher's exact tests were used for categorical comparisons. Pearson or Spearman correlation coefficients were calculated to assess relationships between salivary parameters and erosion severity scores. Multiple logistic regression analysis was performed to identify independent predictors of dental erosion. Statistical significance was set at p < 0.05.

## Results:

All 120 participants completed the study protocol. The intra-examiner reliability for BEWE scoring was excellent, with a kappa value of 0.89. The demographic and baseline characteristics of the study population are presented in Table 1 There were no statistically significant differences between the GERD and control groups in terms of age (p = 0.782), sex distribution (p = 0.714), number of teeth present (p = 0.198), brushing frequency (p = 0.567), or acidic beverage consumption (p = 0.562), indicating appropriate matching between groups. As expected, the GERD group demonstrated significantly higher GerdQ scores compared to controls (p < 0.001). Salivary parameters are summarized in Table 2 GERD patients exhibited significantly lower unstimulated salivary pH (6.32 ±0.41) compared to controls (6.89 ±0.34, p < 0.001). Similarly, stimulated salivary pH was significantly reduced in the GERD group (6.78 ±0.38) relative to the control group (7.21 ±0.31, p < 0.001). Quantitative buffering capacity (final pH) was also significantly lower in GERD patients (4.86 ±0.54) compared to controls (5.62 ±0.47, p < 0.001). Unstimulated salivary flow rate was marginally lower in the GERD group (0.31 ±0.14 mL/min) than in controls (0.36 ±0.12 mL/min), but this difference did not reach statistical significance (p = 0.061). Stimulated salivary flow r ate showed no significant difference between the groups (p = 0.347). Categorical analysis of buffering capacity revealed a significantly different distribution between groups (p < 0.001). A higher proportion of GERD patients exhibited low buffering capacity (36.7%) compared to controls (10.0%), whereas high buffering capacity was more frequent in the control group (60.0%) than in the GERD group (23.3%). Dental erosion prevalence and severity are presented in Table 3 The prevalence of dental erosion (BEWE ≥ 3) was significantly higher in the GERD group (71.7%) compared to controls (28.3%) (p < 0.001). The mean cumulative BEWE score was also significantly greater in GERD patients (7.84 ±4.21) than in controls (3.12 ±2.67) (p < 0.001). Risk stratification based on BEWE scores demonstrated that higher-risk categories were more prevalent in the GERD group (p < 0.001). Specifically, medium- and high-risk erosion scores were observed more frequently among GERD patients, whereas the majority of controls fell into the no-risk c ategory. The most commonly affected surfaces in GERD patients were the palatal surfaces of maxillary anterior teeth and the occlusal surfaces of mandibular posterior teeth. Correlation analysis Table 3 demonstrated significant associations between salivary parameters and erosion severity. Cumulative BEWE scores showed moderate negative correlations with unstimulated salivary pH (r = -0.52, p < 0.001), stimulated salivary pH (r = -0.46, p < 0.001), and buffering capacity (r = -0.58, p < 0.001). Unstimulated salivary flow rate also showed a weak but significant negative correlation (r = -0.24, p = 0.008). Conversely, GERD duration demonstrated a moderate positive correlation with BEWE score (r = 0.49, p < 0.001), and GerdQ symptom score was also positively correlated with erosion severity (r = 0.43, p < 0.001). Multiple logistic regression analysis identified GERD diagnosis (OR = 3.87, 95% CI: 1.82-8.23, p < 0.001), low buffering capacity (OR = 2.94, 95% CI: 1.36-6.35, p = 0.006), and GERD duration > 5 years (OR = 2.41, 95% CI: 1.18-4.92, p = 0.016) as independent predictors of dental erosion. Subgroup analysis within the GERD cohort revealed that patients not receiving proton pump inhibitor therapy had significantly higher BEWE scores (9.42 ±4.38) compared to those on therapy (6.63 ±3.72, p = 0.007). Additionally, these patients demonstrated significantly lower unstimulated salivary pH (6.14 ±0.43 vs 6.46 ±0.36, p = 0.003).

## Discussion:

The current investigation showed that patients with gastroesophageal reflux disease have very low salivary pH and buffering capacity than healthy matched subjects and that this changes are significantly correlated with the occurrence and intensity of dental erosion. These results disqualify the null hypothesis and give detailed evidence of the incorporation of salivary assessment into the dental management procedure of GERD patients. The result of the considerably reduced unstimulated salivary pH of the GERD group (6.32 versus 6.89) is in line with the expected outcomes of prolonged contact with gastric acid on the oral environment. The retrograde intraprostatic flow of very acidic gastric contents in the mouth cavity is a direct assault on salivary pH homeostasis and recurrent acid application may overwhelm the neutralizing ability of saliva leading to a permanently low baseline pH [17]. It is this observation that supports previous reports of low oral pH in reflux patients using intraoral pH measurements which revealed that GERD patients have more and longer periods of oral pH depression below the critical level of 5.5 which is thermodynamically favorable to enamel demineralization [18]. According to clinical viewpoints, the diminished buffering capacity of the GERD group is of particular importance. Salivary buffering capacity is the functional capacity of saliva to oppose acidic pH shifts and neutralize the effects of acid challenge by replenishing the neutral conditions and is largely regulated by the bicarbonate buffer system of stimulated saliva and the phosphate buffer system of unstimulated saliva [19]. The noted decrease in buffering capacity in patients with GERD could be due to chronic depletion of the bicarbonate store as a result of repeated neutralization of the refluxed gastric acid, a similar phenomenon as metabolic buffering depletion in acid-base physiology. Past research has established that chronic exposure to acids can modify the content of the secretions of salivary glands, which might influence the buffering constituent concentration [20]. The fact that the salivary buffering capacity correlates very negatively with the BEWE erosion scores (r = -0.58) is quantitative evidence that the worse buffering capacity, the worse the erosive tooth wear is in GERD patients. This interaction is biologically plausible: as long as the buffering capacity is not high enough to neutralize the acids as soon as they reach the tooth surface, the time of being exposed to critical PH levels is extended leading to more mineral being dissolved in each exposure to acid episode [21]. The long-term consequence of these years of reflux disease of cumulative acid exposure is the development of increasingly severe erosive lesions, which is in line with the high positive correlation of the years of GERD with the severity of erosion, as shown in the current study. The prevalence of 71.7% in the GERD group is significantly higher than the rates in the general population as well as the upper range of numbers in other studies of GERD-erosion. It has the pathognomonic feature of characteristic distribution pattern, i.e., the most frequent appearance on the palatal surfaces of maxillary anterior teeth, as well as the direction of the regurgitated gastrointestinal acid flows, which strike the palatal surfaces of enamel first and most directly in case of reflux episodes [22]. The pattern of distribution is a significant diagnostic indicator to dental practitioners who may be the initial healthcare practitioner to diagnose undiagnosed GERD by observing typical erosion patterns during routine dental check-ups [23]. The results that the use of proton pump inhibitors is related to lower BEWE scores and higher salivary pH, in the GERD subgroup, has significant clinical implications. PPIs lower the amount of gastric acid by blocking irreversibly the production of gastric acid through hydrogen-potassium adenosine triphosphatase system on parietal cell surface, consequently raising the pH of refluxate coming into the mouth [24]. Consistency of the protective effect of PPI therapy on the severity of dental erosion is based on the assumption that refluxate acidity is a key factor in determining dental erosion risk and that pharmacological acid inhibition can avert dental effects. Nevertheless, the erosion remained high amongst the PPI-treated patients (60.3%), which suggests that acids suppression does not prevent the risk of erosion on its own and the dental prevention measures should be used in addition to the dental treatment [25]. The similarity of the salivary flow rate across the groups, though consistent with others that have been reported before, is a contrast to other studies that have also reported an augmentation in the stimulated salivary flow in GERD patients as an element of the esophagosalivary reflex [26]. It is a reflex mechanism that is mediated by vagal afferents in response to the exposure of the esophagus to acid, which will result in augmented salivary secretion, which will lead to the elimination of esophagal acid. The lack of flow rate difference observed in the current study can be attributed to the heterogeneity of reflex responsiveness among individuals, the confounding effect of PPI treatment that diminishes the strength of the acid stimulus, or cross-sectional study design that only consists of one time-point measurement of the flow response of an actual reflux episode [27]. The multiple logistic regression models has recognized three independent predictors of dental erosion GERD diagnosis, low buffering capacity and a disease duration above five years to offer a rough risk assessment model that can be used in clinical practice. Of them, there was the greatest association with GERD diagnosis (OR = 3.87), which proves that reflux disease itself is the most significant risk factor. The separation of the effect of low buffering capacity on erosion at constant GERD status emphasizes the modulating effect of the personal salivary features on the erosion vulnerability of patients with relative levels of acid exposure [28]. The observation confirms the idea that salivary buffering capacity measurement may be a clinically significant biomarker of the presence of GERD patients at greatest risk of erosive dental injury, which can be utilized to deliver preventive measures. There are a number of limitations on which these findings are to be interpreted. The cross-sectional design does not allow the construction of the cause-effect relationship between the change in the salivary parameters and the erosion progression. Salivary pH and buffering capacity measurements are a single time-point measure that is not necessarily representative of dynamic changes during and after reflux events. The nutritional history basis was based on self-reported data on a questionnaire and that is prone to recall bias and may not appropriately adjust the confounding impact of extrinsic sources of acids [29]. Although clinically useful and valid, BEWE index does not distinguish erosion active and arrested or erosion of various etiologies. Prospective longitudinal studies in the future which involve repeat salivary determinations, chronic intraoral pH testing, along with comprehensive analysis of the diet would enhance the basis of causation [30]. GERD-associated alterations in salivary composition and function play a pivotal role in the development of dental erosion, underscoring the need for coordinated interdisciplinary preventive and management strategies [31].

## Conclusion:

Patients with gastroesophageal reflux disease exhibit significantly reduced salivary pH and buffering capacity, contributing to a persistently acidic oral environment. This impairment is strongly associated with increased prevalence and severity of dental erosion, highlighting GERD as a major risk factor. Salivary assessment may serve as a valuable tool for early identification and preventive management of erosion risk in these patients.

## Figures and Tables

**Table 1:** Demographic and baseline characteristics of study participants

**Characteristic**	**GERD Group (n = 60)**	**Control Group (n = 60)**	**p-value**
Age (years), Mean ± SD	41.3 ± 9.7	40.8 ± 10.2	0.782
Sex (Male/Female)	28/32	26/34	0.714
Number of teeth present, Mean ± SD	27.4 ± 1.8	27.8 ± 1.6	0.198
Brushing frequency (≥2x/day), n (%)	38 (63.3%)	41 (68.3%)	0.567
Acidic beverage consumption (≥1x/day), n (%)	22 (36.7%)	19 (31.7%)	0.562
GERD duration (years), Mean ± SD	4.8 ± 2.9	-	-
Current PPI use, n (%)	34 (56.7%)	-	-
GerdQ score, Mean ± SD	10.4 ± 2.1	3.2 ± 1.4	< 0.001

**Table 2:** Comparison of salivary parameters between GERD and control groups

**Parameter**	**GERD Group (n = 60) Mean ±SD**	**Control Group (n = 60) Mean ±SD**	**p-value**
Unstimulated flow rate (mL/min)	0.31 ±0.14	0.36 ±0.12	0.061
Stimulated flow rate (mL/min)	1.24 ±0.42	1.31 ±0.38	0.347
Unstimulated salivary pH	6.32 ±0.41	6.89 ±0.34	< 0.001
Stimulated salivary pH	6.78 ±0.38	7.21 ±0.31	< 0.001
Quantitative buffering capacity (final pH)	4.86 ±0.54	5.62 ±0.47	< 0.001
**Categorical buffering capacity:**			
High, n (%)	14 (23.3%)	36 (60.0%)	
Moderate, n (%)	24 (40.0%)	18 (30.0%)	< 0.001
Low, n (%)	22 (36.7%)	6 (10.0%)	

**Table 3:** Dental erosion prevalence, severity and correlations with salivary parameters

**Panel A: Erosion Prevalence and BEWE Scores**	**GERD Group (n = 60)**	**Control Group (n = 60)**	**p-value**
Erosion prevalence (BEWE ≥3), n (%)	43 (71.7%)	17 (28.3%)	<0.001
Cumulative BEWE score, Mean ± SD	7.84 ±4.21	3.12 ± 2.67	<0.001
**BEWE Risk Level:**			
No risk ( ≤ 2), n (%)	17 (28.3%)	43 (71.7%)	
Low risk (3-8), n (%)	22 (36.7%)	13 (21.7%)	<0.001
Medium risk (9-13), n (%)	14 (23.3%)	3 (5.0%)	
High risk (≥14), n (%)	7 (11.7%)	1 (1.7%)	
**Panel B: Correlations (Spearman's rho) with BEWE score**	**r**	**p-value**	
Unstimulated salivary pH	-0.52	<0.001	
Stimulated salivary pH	-0.46	<0.001	
Quantitative buffering capacity	-0.58	<0.001	
Unstimulated flow rate	-0.24	0.008	
GERD duration (years)	0.49	<0.001	
GerdQ symptom score	0.43	<0.001	
